# Finding missed cases of familial hypercholesterolemia in health systems using machine learning

**DOI:** 10.1038/s41746-019-0101-5

**Published:** 2019-04-11

**Authors:** Juan M. Banda, Ashish Sarraju, Fahim Abbasi, Justin Parizo, Mitchel Pariani, Hannah Ison, Elinor Briskin, Hannah Wand, Sebastien Dubois, Kenneth Jung, Seth A. Myers, Daniel J. Rader, Joseph B. Leader, Michael F. Murray, Kelly D. Myers, Katherine Wilemon, Nigam H. Shah, Joshua W. Knowles

**Affiliations:** 1 Center for Biomedical Informatics Research, Stanford University, Stanford, CA USA; 2 Department of Computer Science, Georgia State University, Atlanta, GA USA; 3 Cardiovascular Medicine and Cardiovascular Institute, Stanford University, Stanford, CA USA; 4 Atomo, Inc, Austin, TX USA; 5 Perelman School of Medicine at the University of Pennsylvania, Philadelphia, PA USA; 6 The FH Foundation, Pasadena, CA USA; 7 Geisinger Health System, Genomic Medicine Institute, Forty Fort, PA USA; 8 Center for Genomic Health, Yale University, New Haven, CT USA; 9 Stanford Diabetes Research Center, Stanford, CA USA

**Keywords:** Translational research, Health care

## Abstract

Familial hypercholesterolemia (FH) is an underdiagnosed dominant genetic condition affecting approximately 0.4% of the population and has up to a 20-fold increased risk of coronary artery disease if untreated. Simple screening strategies have false positive rates greater than 95%. As part of the FH Foundation′s FIND FH initiative, we developed a classifier to identify potential FH patients using electronic health record (EHR) data at Stanford Health Care. We trained a random forest classifier using data from known patients (*n* = 197) and matched non-cases (*n* = 6590). Our classifier obtained a positive predictive value (PPV) of 0.88 and sensitivity of 0.75 on a held-out test-set. We evaluated the accuracy of the classifier′s predictions by chart review of 100 patients at risk of FH not included in the original dataset. The classifier correctly flagged 84% of patients at the highest probability threshold, with decreasing performance as the threshold lowers. In external validation on 466 FH patients (236 with genetically proven FH) and 5000 matched non-cases from the Geisinger Healthcare System our FH classifier achieved a PPV of 0.85. Our EHR-derived FH classifier is effective in finding candidate patients for further FH screening. Such machine learning guided strategies can lead to effective identification of the highest risk patients for enhanced management strategies.

## Introduction

Familial hypercholesterolemia (FH) is an autosomal dominant condition with an estimated prevalence of approximately 1 in 250,^1^ making it the among the most common morbid monogenic disorders. Lifelong elevation of low-density lipoprotein cholesterol (LDL-C) in individuals with FH cause up to a 20-fold excess risk of atherosclerotic cardiovascular disease (ASCVD) versus those with normal LDL-C levels.^2,3^ Importantly, the risk of ASCVD can be largely ameliorated through early identification and treatment with lipid-lowering therapies.^1,4–6^ In addition, because FH is highly penetrant, once an individual with FH is identified, cascade screening of relatives has been shown to be highly cost-effective in reducing excess morbidity in family members.^2,6–8^ The importance of differentiating FH from other causes of high LDL-C is reflected by guidelines from multiple national and international organizations, with FH-specific recommendations covering diagnosis, treatment and cascade screening.^1,5,6^

Despite the morbidity and mortality associated with FH and the clear benefits of timely management, it is estimated that less than 10% of persons with FH in the US have been diagnosed,^1^ with the identification of index FH cases (probands) as a major bottleneck. Currently, guidelines recommend the application of diagnostic criteria (e.g., Dutch Lipid Clinic Network (DLCN) or Simon-Broome) in adults for which there is high clinical suspicion, which is usually based on untreated LDL-C values >190 mg/dl plus a positive family history of early onset ASCVD.^1,5,6^ However, there are significant limitations to this approach. For instance, this strategy is non-specific: While high LDL-C is a cardinal feature of FH, less than 5% of adults with an LDL-C > 190 mg/dl will be found to harbor a causal FH gene mutation.^3^ In addition, this strategy largely relies on the availability of untreated LDL-C values and adequate family history information, either/both of which are often unavailable to the healthcare provider.

We sought to develop a classifier that could prioritize individuals within a healthcare system to undergo further evaluation for FH, thereby enhancing the efficiency of case identification. Machine-learning algorithms can analyze large datasets and determine combinations of variables that consistently classify or predict a certain outcome.^9^ Such models have been widely applied in non-medical fields^10^ with nascent but promising use in medicine.^11,12^ Widespread adoption of EHRs has led to large collections of patient-level data being available for the development of such algorithms.

As part of the FH Foundation′s FIND (Flag, Identify, Network, Deliver) FH initiative, here we report the development and internal validation of a supervised machine-learning algorithm to identify probable FH cases based on EHR data from Stanford Health Care as well as the external validation on this classifier using EHR data from the Geisinger Healthcare System. The performance of the classifier, which achieves a PPV of >0.8 across two independent datasets, and the resulting reduction in testing cost as well as case-finding burden, suggests that application of this classifier could lead to increased efficacy of targeting these high-risk patients for enhanced evaluation and intervention.

## Results

### Study design

**Fig. 1:**
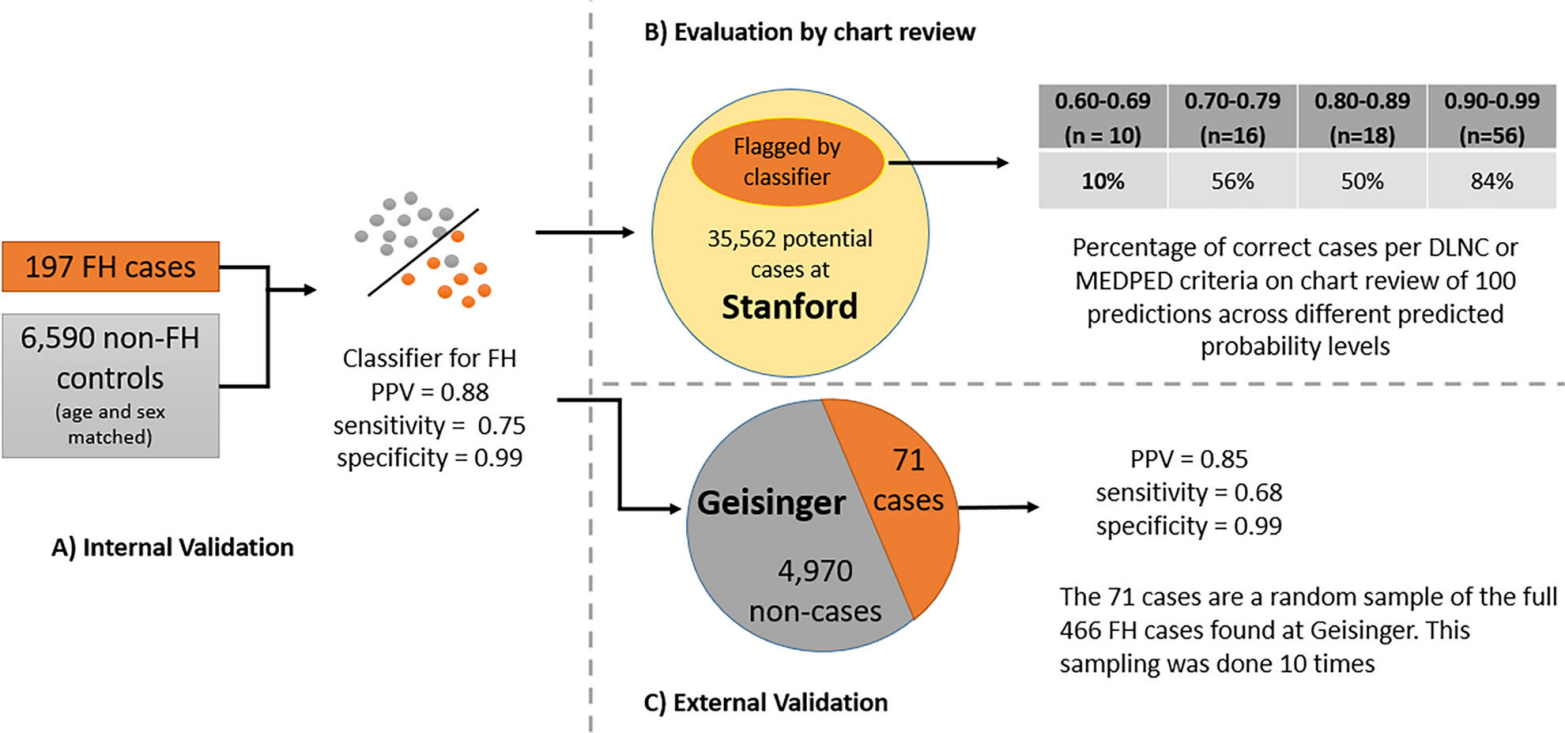
Classifier building followed by internal and external evaluation as well as evaluation via chart review for EHR-based FH case identification

Our classifier was built using both structured and unstructured EHR data from Stanford as described in the methods. We confirmed clinical utility at the local site via manual chart review of patients flagged by the classifier and validated at an independent site (Geisinger) with genetically confirmed FH cases (Fig. 1).

As described in the methods, we developed a random forest classifier^13^ due to their resistance to overfitting, model interpretability, and ranking of important features. To quantify the performance of the classifier, we used common information retrieval metrics,^14^ namely positive predictive value (or precision), sensitivity (or true positive rate), specificity (true negative rate) and F1 score (or F-Measure). Our classifier obtained a positive predictive value (PPV) of 0.88 and sensitivity of 0.75 (Fig. 1a and Table 1) on a held-out test-set. We also report the area under the receiver operator curve (AUROC) and the area under the precision-recall curve (AUPRC), which is more informative for low prevalence outcomes.^15^ We included F1 score and AUPRC as part of our evaluation metrics since the F1 score summarizes model performance at a specific probability threshold, in contrast, the AUPRC value summarize the performance of a model across all possible thresholds. Thus, F1 and AUPRC provide complementary information. In the supplementary materials, under Random Forest Classifier Error Analysis, we provide the classifier error analysis and AUROC and AUPRC plots for clarity.

**Table 1:** Classifier performance at internal and external sites

	Internal evaluation (Stanford)	External evaluation (Geisinger)
AUROC	0.94	0.94 (0.003)
AUPRC	0.71	0.68 (0.054)
PPV	0.88	0.85 (0.002)
Sensitivity	0.75	0.68 (0.002)
Specificity	0.99	0.99 (0.001)
F1 Score	0.81	0.75 (0.004)

For the internal evaluation, the table reports performance metrics on a held-out test-set. For the external evaluation, the table reports the average performance over 10 iterations of classifying randomly sampled 71 cases and 4970 non-cases at 1:70 prevalence, which mirrors expected prevalence in a lipid clinic. The numbers in the in parentheses are standard deviations for each metric

### Evaluation via chart review

The classifier outputs the probability of each patient being a case. Given the use case of the classifier—which is to drive screening and further evaluation of flagged patients—we selected 100 patient records held out from the training data from multiple bins of the classifier output ranging from probability 0.99–0.90, 0.89–0.80, 0.79–0.70, and 0.69–0.60 (Fig. 1b). We reviewed more charts from the high probability cases, and fewer of those that have a low chance of being a case to get the greatest granularity on the predictions in the probability group that is most likely to be put into practice.

**Fig. 2:**
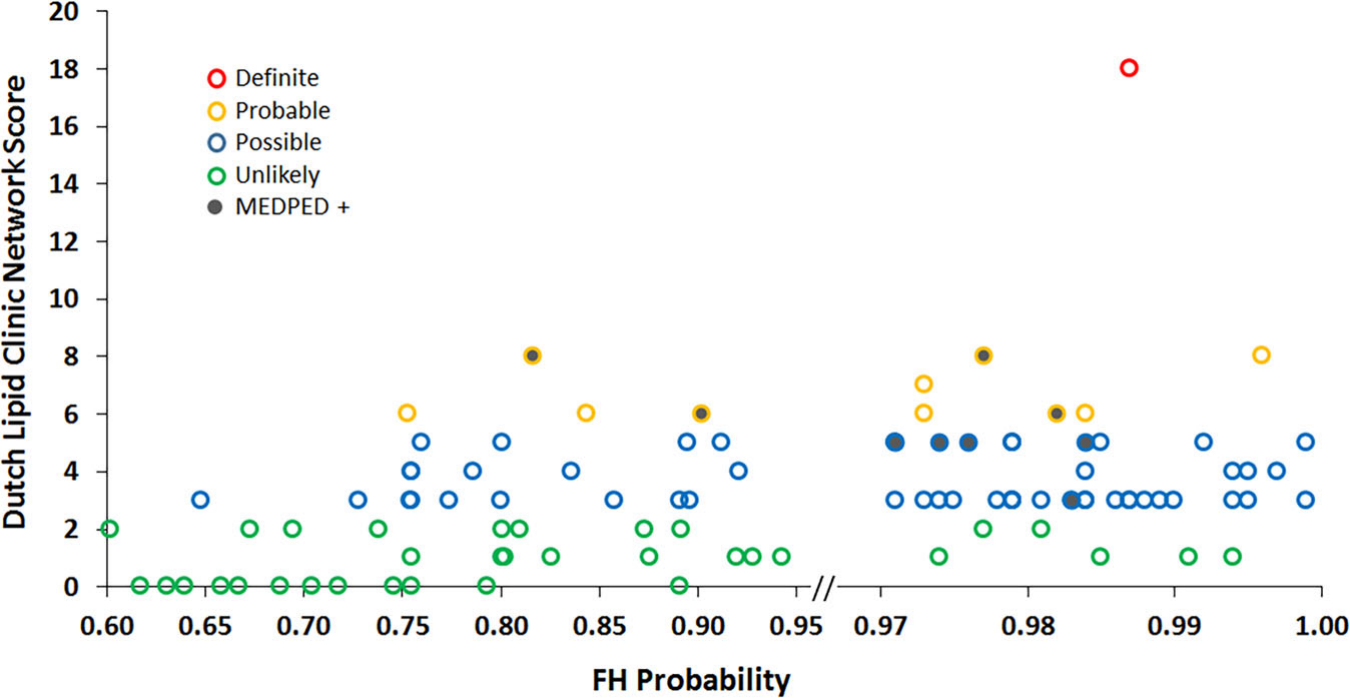
Distribution of FH cases according to probability assigned by the random forest classifier as arbitrated by independent chart review at Stanford

Of the 56 predictions with a probability score of 0.99-0.90, 39 have a DLCN score of 3–5 (“possible” FH) and 5 of these would meet MEDPED criteria, 7 have a DLCN score of 6–8 (“probable” FH) and 3 of these would meet MEDPED criteria and 1 has a DLCN score > 8 (“definite” FH). In other words, 47/56 have a DLCN score of >=3 or are MEDPED positive (84%). In contrast: only 9/56 have a DLCN score of 1 or 2 (unlikely)(16%). As expected, the rate of likely cases diminishes in lower probability bins especially in those with a probability score < 0.7 (graphically represented in Fig. 2 and Supplementary Table 6).

### External validation

We validated our classifier and its ability to detect FH patients by evaluating it on an independent dataset from Geisinger (Fig. 1c). This evaluation is performed by providing a set of 71 cases and 4970 controls to the Stanford classifier, ten times. We sample the 71 cases from the full set of 466 cases provided by Geisinger (see dataset details in Methods). Directly classifying the Geisinger dataset with its native 466:5000 case to non-case ratio would lead to overly optimistic estimates of performance.

Table 1 shows classifier performance in terms of the PPV, specificity, and sensitivity as well as the AUROC, AUPRC, and F1 score. As expected, there is a slight drop in PPV, AUROC, and AUPRC, given the different operational practices between institutions (coding, labs, clinical narratives). Limiting the analysis to just the genetically confirmed cases has an F1 score of 0.82. The results show that the classifier trained with Stanford data has good external validity in identifying FH patients at a different institution.

For completeness, we also built a classifier using Geisinger data (which had 0.88 PPV, 0.74 sensitivity, and 0.99 specificity) and tested its ability to classify Stanford patients. This classifier achieved a PPV of 0.83, sensitivity of 0.66 and specificity of 0.99.

Table 2 showcases the top 20 features used by the random forest to assign a probability score to each patient for having FH. These features are a vital part of the interpretability aspect of the random forest models as they can be traced for every patient assigned by the algorithm. The classifier uses a combination of laboratory tests, text mentions, diagnosis codes and prescriptions as the top features with many of them being related to cholesterol levels and hyperlipidemia diagnosis. The list includes text mentions about the disease (Lipid, Triglycerides) and possible lifestyle adjustments (Red Meat), demonstrating the importance of using statistical models on all the patient data available. One thing to note is that all the laboratory tests selected as informative features correspond to the high and very high bins created during feature engineering, which capture the higher than usual total cholesterol and LDL-C measurements.

**Table 2:** Top 20 features in the classifier that flag patients with FH

#	Feature_ID	Source	Feature explanation, and source
1	text:40094263	Unstructured	Mention of LDL cholesterol in doctors′ notes
2	lab:3027114:BIN5	Structured	*Very high*, Cholesterol value in Serum or Plasma. (*note: the ranges for very low, low, in-range, high, very high are learned during model training*)
3	text:457658075	Unstructured	Mention of a visit to a Cardiology clinic
4	cond:448359416	Structured	A diagnosis code of Paroxysmal supraventricular tachycardia
5	drugEx:15459583	Structured	A prescription of atorvastatin
6	lab:3028288:BIN4	Structured	*High*, calculated LDL cholesterol in Serum or Plasma
7	drugEx:15264753	Structured	A prescription of ezetimibe
8	text:40372345	Unstructured	Mention of ‘Red meat′ (indicative of diet conversations)
9	lab:3028288:BIN5	Structured	*Very High*, calculated LDL cholesterol in Serum or Plasma
10	lab:3009966:BIN4	Structured	*High*, LDL cholesterol in Serum or Plasma by Direct assay
11	text:42897633	Unstructured	Mention of ‘Lipid′ in doctors notes
12	lab:3025839:BIN5	Structured	*Very High*, Triglycerides in Serum or Plasma
13	text:45957223	Unstructured	A mention of ‘Triglycerides’
14	drugEx:15108133	Structured	A prescription of rosuvastatin
15	cond:448369299	Structured	Mixed hyperlipidemia
16	cond:448276299	Structured	Other and unspecified hyperlipidemia
17	drugEx:13070462	Structured	A prescription of Metoprolol
18	text:457636305	Unstructured	A mention of Rosuvastatin
19	lab:3027114:BIN4	Structured	*High*, Cholesterol value in Serum or Plasma
20	text:4230588	Unstructured	A mention of ‘Cytologic’

## Discussion

The role of risk-stratification models and predictive algorithms to identify “high-risk” patients is well-established in clinical medicine. The 2013 American College of Cardiology/American Heart Association Omnibus calculator to identify non-FH patients who would benefit from statin initiation for primary prevention of ASCVD stands as a key example of a risk-stratification algorithm used in common practice.^16^

The advent of machine learning approaches presents an opportunity to leverage EHR data to develop risk-stratification and predictive models at scale.^17^ Notably, the ability of machine-learning algorithms to be trained on both structured and unstructured EHR data—such as free text—allows the use of variables that may not be considered in traditional settings. Machine-learning derived predictive models may be particularly suited to address care gaps for treatable conditions that have traditionally been underdiagnosed.

It is estimated that only ~10% of patients with FH are diagnosed in the United States.^7^ After an index case with FH is identified, cascade screening is highly effective in identifying affected family members. An initial diagnosis of FH generally involves the use of the MEDPED criteria, the UK Simon-Broome register criteria, and the DLCN criteria. These clinical criteria require manual imputation of certain variables: patient history including ASCVD events and pretreatment lipid levels, physical examination findings such as tendon xanthomas and arcus cornealis, family history details including LDL-C values of first-degree relatives, and results of patient genetic testing. The utility of these criteria is unclear given the real-world challenges in obtaining detailed family histories, low prevalence of variables such as physical exam findings like tendon xanthomas or elevated lipid levels and ASCVD events in relatives.^18–21^

Therefore, there is a strong need to develop better approaches to screen for FH. Given the widespread use of EHRs, a machine-learning based approach could increase the rate of index FH case identification at low cost. Indeed, among the patients flagged by our random forest classifier for whom chart review confirmed a likely diagnosis of FH, only 3 had been clinically diagnosed with FH. The formal diagnosis of FH should lead to a greater focus on effective management and more intensive therapy for LDL-C reduction. Although it is not required for diagnosis, genetic testing is a useful component of making a diagnosis of FH.^22^ Given the low prevalence of the condition, routine universal genetic testing of everyone with a high LDL-C is low yield and inefficient. However, knowledge of genetically defined subgroups within cohorts of clinically diagnosed FH cases is expected to ultimately drive differential management strategies, and thereby increase the value of genetic testing in this condition.

Recently, Safarova et al.^23^ described developing an automated process to score patients using the DLCN Criteria using both structured and unstructured EHR data. This process uses textual reports to determine the family history of having a first-degree relative with hypercholesterolemia or premature ASCVD, and to determine the presence of characteristics such as tendon xanthomas and corneal arcus. The quality of the text extraction is validated by reviewing 20 randomly selected charts to have sensitivity and specificity of 97 and 94%, with positive and negative predictive values at 94 and 97%, respectively. Subsequently, the natural language processing (NLP) extracted variables and other structured data elements are used to computationally “apply” the modified DLCN criteria to obtain a score.

Our effort has a fundamentally different approach. We learn a classifier that directly discriminates FH cases from non-cases without computing DLCN criteria as an intermediate. Of the cases our classifier flags, we evaluated them to be true or false based on several criteria, including genetic testing at an independent site. Our random forest classifier demonstrated good positive predictive value and sensitivity upon application to an unseen internal test dataset (ppv 0.88, sensitivity 0.75) and an external Geisinger EHR dataset (ppv 0.85, sensitivity 0.67) including those with genetically confirmed FH. These findings point to the external validity and overall potential utility of our EHR-based classifier to screen for patients with a high probability of FH. In a similar vein, Bastarache et al.^24^ have shown that it is possible to build phenotype risk score for identification of patients with underrecognized Mendelian disease patterns (though not for FH) by leveraging EHR data.

It is also natural to ask why not use “deep learning”. Given the small data size at hand and the chance for a deep neural network to overfit, coupled with the desire to have an interpretable model that would generalize across multiple sites, we used a simpler modeling approach.^25,26^

In summary, the use of a classifier to detect putative cases of FH from the EHR allows the identification of patients who have a substantial probability of having the condition. Clinicians can then perform targeted evaluation to confirm index FH cases, place referrals to appropriate subspecialty clinics for additional evaluation for FH, and eventually initiate therapy and cascade screening. Additionally, developing machine-learning algorithms may allow the identification of novel predictive variables. For instance, the top 20 predictive concepts in our random forest classifier include variables not used in the traditional FH clinical criteria, such as the diagnosis codes for paroxysmal supraventricular tachycardia and triglyceride levels (Table 2).

Our work has certain limitations. For the Stanford test dataset, we used an (estimated) FH prevalence of 1:70 in the test-set, if the real-life prevalence of FH cases in a lipid clinic is drastically different the classifier performance will differ. Measuring downstream outcomes such as the overall rates of FH diagnosis, cascade screening, ASCVD events or survival would require longitudinal observation after implementation of this algorithm and was not addressed in this study. While we have demonstrated that this classifier ported well to data from another health system, it is possible that we might see a performance increases if we used the data from both sites in order to train our classifier. We did not train using pooled data because doing so would not allow the assessment of external validity of the classifier. Finally, our sample of positive FH patients is relatively small (*n* = 197 at Stanford); and as with most machine learning approaches, having more training data would probably build a better classifier. We anticipate continuously refining our classifier as newly diagnosed cases accrue.

The ultimate utility of any screening test must be considered in the context of its cost-effectiveness. While we report multiple metrics of performance. In the current use case, due to the expense of manual chart review and follow on genetic testing, we aim for better PPV because it quantifies the frequency with which predictions are relevant or ‘worth following up′. If the cost of the follow-up action (chart review, and genetic testing) become negligible in the future, it would make sense to aim for higher recall at the expense of a lower PPV. For example, if FH occurs at a probability of 1 in 70 in a cardiology clinic with costs of $1000 to do genetic counseling and testing, and 15 min to apply the screening criteria, for each case found we would need to spend roughly $70,000 in genetic testing and 1050 min of clinician time. However, after applying EHR-based screening, the chance that an individual flagged by our algorithm has FH is 8 out of 10. As a result of this massive chance in post-test prevalence, the cost to find one new case drops to $1429 in genetic counseling and testing, and 21.4 min of clinician time. Therefore, compared to the implementation of universal genetic testing or clinical criteria-based screening, the economics of EHR-based detection of FH through machine-learning are extremely favorable and can massively improve the ability of a health system to find patients at risk. We believe the use of supervised learning to build a classifier that finds undiagnosed cases of FH is a compelling example of machine learning that matters.^27^ As a next step, we are working on deploying the model in a clinical setting, at Stanford Healthcare and at additional sites in partnership with the FH Foundation.

In conclusion, we have demonstrated that a supervised machine learning approach to building a classifier for finding patients that might have FH using EHR data is feasible with a positive predictive value of 0.88, sensitivity of 0.75 and specificity of 0.99. We validated our classifier by classifying 35,562 patients and reviewing predictions across a range of probability scores via chart review, and by applying established criteria, such as DLCN and MEDPED criteria, to determine the likelihood of the patients flagged by our classifier to have FH. We used unseen FH and non-FH patient data from the Geisinger Healthcare System to demonstrate external validity of the classifier. Compared to universal genetic testing or clinical criteria-based screening of all comers, the use of EHR-based detection of FH through machine-learning can massively improve the ability of a health system to find patients at risk of FH. Such case finding is particularly relevant because once a case is found, proven efficacious interventions already exist that can prevent catastrophic cardiovascular events; furthermore, that case can be used to ‘cascade′ to find multiple other cases within an extended family. Applied broadly, using our classifier to screen using EHRs could identify many thousands of the undiagnosed patients with FH and lead to more effective therapy and screening of their families.

## Methods

### Study design

**Fig. 3:**
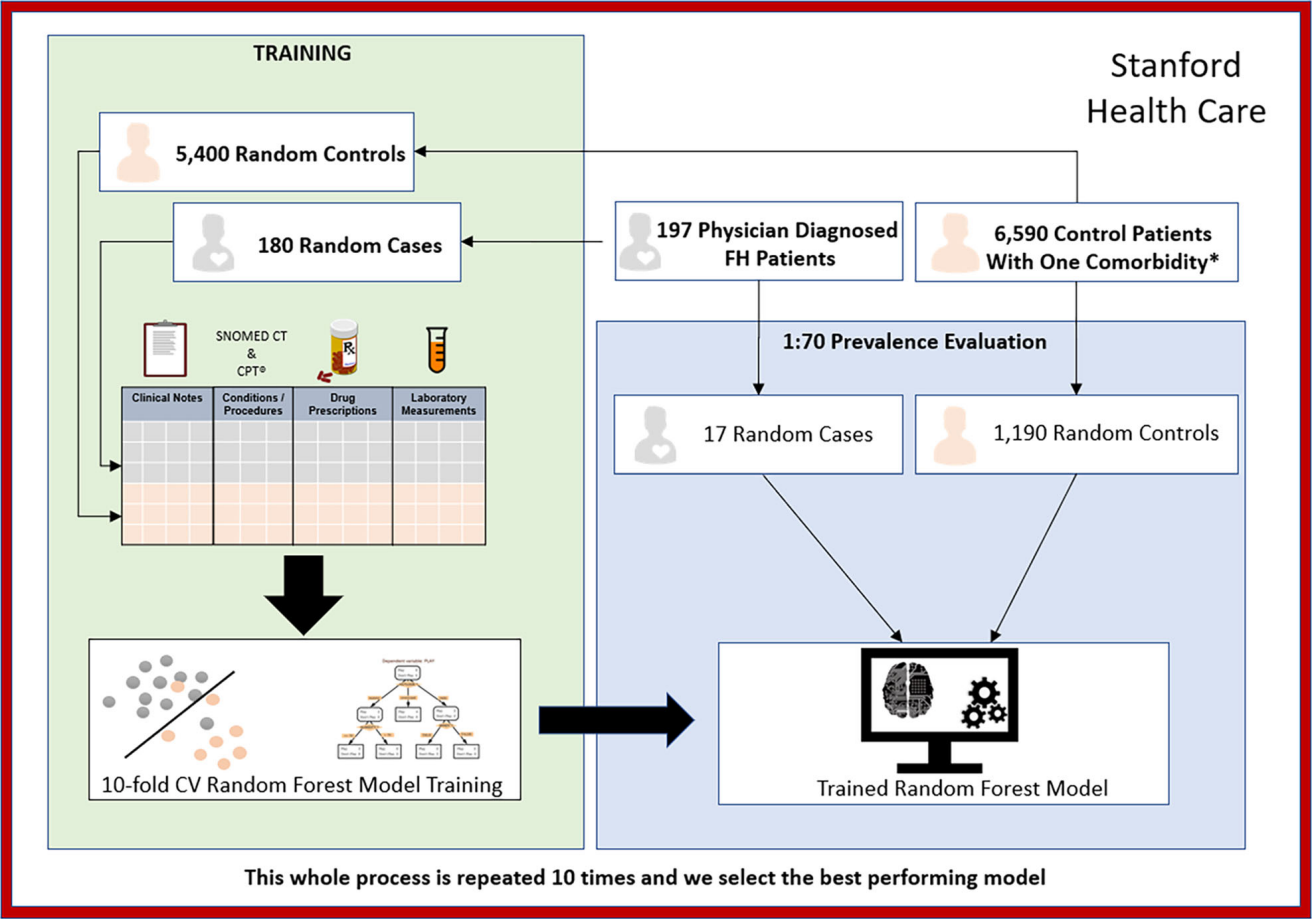
Learning and testing setup for the Stanford FH classifier. * One comorbidity from the following: hypertension, coronary atherosclerosis (CAD), dyslipidemia, myocardial infarction; and had no history of nephrotic syndrome, or obstructive (cholestatic) liver disease

Our classifier was built using both structured (e.g., labs, procedures, diagnostic codes) and unstructured (e.g., text from clinical notes and radiology reports) EHR data from Stanford and validated with data from an independent site (Geisinger) including a subset of genetically confirmed cases (Fig. 1). We perform our work using data in the OMOP common data model (OMOP-CDM). The building of the classifier is shown in Fig. 3.

### A common data model and community tools

The Observational Health Data Sciences and Informatics (OHDSI) is a world-wide collaborative which features over 140 collaborators in 16 countries comprised of healthcare industry leaders, clinical researchers, computer scientists, and biostatisticians. OHDSI′s vision is to improve health by empowering a community to collaboratively generate evidence that promotes better health decisions and better care.^28^ The community has developed both a common data model (CDM), as well as a standard vocabulary for consistent representation of EHR data across sites. The CDM is comprised of 39 standardized tables that have gone through a refinement process over 5 iterations initially through the OMOP initiative.^29^ Approximately 84 sites have converted their local data into the common OHDSI CDM including both clinical and claims, totaling over 600 million patients. APHRODITE^30^ is an open source software package for building phenotype models using data in the OMOP CDM.

### Dataset, Stanford Health Care

This dataset integrates patient data from Stanford Children′s Health (SCH) and Stanford Health Care (SHC) hospitals (Fig. 3). We used data from 3.1 million patients, which includes 70 million clinical notes including pathology, radiology and transcription reports, over 90 million coded diagnoses and procedures, 269 million laboratory tests and 59.3 million medication orders. From the clinical notes, we extracted over 7.8 billion clinical terms using a custom text processing workflow which recognizes present, non-negated mentions of terms from 22 clinically relevant ontologies (SNOMED, Human Disease Ontology, MedDRA among others). Each term is mapped to an UMLS CUIs while making sure that negative term mentions are flagged using NegEx regular expressions.^31^ More details about the text processing pipeline used can be found in ref. ^32^ The data are mapped to the OHDSI CDM version 5.0 using vocabulary v5.0 11-MAR-16. Newer versions might change the CDM structure, but the updates are backwards compatible. In addition, the Aphrodite package will be updated with CDM releases to make older phenotype models backwards compatible.

### Classifier building

We used the APHRODITE, and other R packages designed by the OHDSI community, to extract all patient data, build patient feature matrices, and train machine learning models from the data. As features, we used the count of times a code, lab, drug prescription was found on the patient′s record. We normalized features by length of patient follow-up in years, removed features found in less than 10% patients, and excluded text mentions that are not medical terms. Finally, we binned the lab values for a given lab test in five discrete bins (very low, low, in-range, high, very high), which allowed us to handle extreme lab values that are characteristic of patients with potential FH. The bins are determined by acquiring the minimum and maximum values for each specific lab and then splitting the range in five equal bins.

To build our classifier, we used 197 known FH patients from Stanford (Figs. 1a and 3). The demographics tables for these cohorts are found in the Supplementary Appendix section (Supplementary Table 1). The FH patients were followed at an FH-specific clinic and had been diagnosed as probable or definite FH using existing diagnostic criteria including genetic testing information when available.^5,33–36^ The application of these criteria is done manually and takes upwards of 15–20 min of clinician time. Among these patients, the average pretreatment LDL-C was 258 mg/dl. Sixty-six were known to have a causal genetic variant in *LDLR* or *APOB*, 26 had undergone genetic testing that did not identify a causal mutation and 105 had not undergone genetic testing. There were no patients that were found to have causal PCSK9 variants. This is not unexpected as <1% of FH patients harbor causal mutations in PCSK9.

To ensure relevance of our classifier for use on patients at a higher risk for ASCVD for discerning between FH cases and similar non-FH cases “in the wild”,^37,38^ and to ensure that trivial classification (e.g., healthy 25-year olds classified as non-FH) does not produce optimistic results, we limited our non-cases (“controls”) to patients that: (1) had more than one visit at Stanford; (2) had at least one visit within the last 2 years; (3) had one comorbidity from the following: hypertension, coronary atherosclerosis (CAD), dyslipidemia, myocardial infarction. We also excluded patients with nephrotic syndrome, or obstructive (cholestatic) liver disease because both result in extremely elevated LDL-cholesterol levels for entirely different reasons and do not convey the same risk of coronary artery disease. Before training the classifier, we matched cases and controls by age, gender, and length of record, in order to avoid trivial classification. On applying these criteria, we had 35,562 patients as potential controls (Figs. 1a, b and 3).

Because the choices about disease prevalence affect classifier performance, it is important to develop the classifier for the specific scenario in which the classifier would be applied. While the population prevalence of FH is approximately 1 in 250 individuals, it is known to be more twice or three times as common in certain clinical settings such as in patients at high-risk of ASCVD or with hypercholesterolemia. In our case, we believe that this classifier would be most useful in flagging individuals within the healthcare system with an enhanced risk of ASCVD, we set a prevalence of 1:70 for evaluation, which is a reasonable estimate given the known prevalence of FH in individuals with hypercholesterolemia or increased risk of ASCVD (Supplementary Table 1).^3^

However, 1:70 is still a severe class imbalance for training a good classifier. Therefore, when training, we use a 1:30 prevalence, essentially upsampling the rare class (i.e., the positive cases) and downsampling the common class. Such upsampling (or downsampling) is a commonly used technique in machine learning with severe class imbalance.^39^ When evaluating the performance of the classifier, we use the 1:70 prevalence which is closest to the environment in which the classifier will be used.

From the total cases (*n* = 197) and controls (*n* = 35,562), we down-sampled the non-FH patients when training the classifier. We split the positive cases into test (*n* = 17) and training (*n* = 180) sets. From the potential controls (non-cases), we selected 5,400 random controls in the training set (setting 1:30 prevalence for the positive class) and selected 1190 for the test-set (setting a 1:70 prevalence for the positive class) (Fig. 2). We trained the classifier using 10-fold cross-validation, on the 1:30 prevalence training set, for parameter tuning. The best performing classifier from this process was tested on the unseen 17 cases and 1190 controls with 1:70 prevalence. We repeated the entire process 10 times (choosing different splits of the train/test sets). We selected the best performing classifier from these ten runs for evaluation by chart review and for external validation.

In previous work,^40^ we built and compared a logistic regression classifier with the random forest classifier. The overall performance was lower than the random forest, and in the current work, we only use the random forest classifier.

### Evaluation via chart review

As shown in Fig. 1b, we applied the random forest classifier to held out patients from Stanford to flag potential FH cases, and evaluated the predictions with manual chart review. We randomly sampled 100 cases from those flagged by the classifier at different probability cutoffs. Our sample contained 56 patients with a probability of being an FH case between 0.99 and 0.90, 18 patients between 0.89 and 0.80, 16 between 0.79 and 0.70, and the remaining 10 from the probability ranges between 0.69 and 0.60 to perform a systematic chart review to assess the predictions at different thresholds. Patients were then scored using the DLCN or MEDPED criteria^1,5^ to determine the relationship between the machine-learning algorithm and these widely used clinical criteria, which are currently used to help inform choices about referral and clinical care. When evaluating the identified patients via chart review, individuals were judged to have FH if they met either DLCN or MEDPED criteria.

The DLCN criteria assigns points for based on clinical findings (e.g., LDL-cholesterol levels, history of coronary artery disease, physical exam), family history and genetic testing results (if known). Based on the score, individuals are categorized as definite, probable, possible or unlikely FH. Those with higher scores are more likely to have a causal mutation identified on genetic testing. The MEDPED criteria are based on age-adjusted cholesterol levels and factor into account family pedigree information (if known) with cutoffs designed to identify individuals who would have a causal mutation found on genetic testing.^33^

### External validation in the Geisinger Healthcare System

The validation dataset contains a subset of the Geisinger data warehouse containing a total of 33,086 patients, with 3 million clinical notes, 32 million laboratory tests, 27 million medication orders. Geisinger investigators provided full NLP extraction of clinical terms using their internal text processing workflow for 5466 patients. The Geisinger data were mapped to the OHDSI CDM version 5.0 using vocabulary v5.0 11-MAR-16. The data contain 466 FH cases that have been diagnosed using the DLCN criteria,^2^ of which 236 FH cases were confirmed by genetic testing.

For external validation, we applied our Stanford classifier to unseen data from Geisinger. Note that directly classifying the external dataset with its native 466:5000 case to non-case ratio would lead to overly optimistic estimates of PPV. Therefore, we used a subset (*n* = 71) of the 466 Geisinger FH cases and controls (*n* = 4970) in a 1:70 ratio, to assess the ability of the classifier trained at Stanford to discriminate true cases from non-cases. We present the average results of the ten evaluation runs along with the standard deviation of the different metrics.

For all patient data, we have complied with all relevant ethical regulations and the study was approved by Stanford University′s institutional review board with waiver of informed patient consent. The model building was performed with de-identified data and only the members of the chart review team were provided with access to medical records. The study only made secondary use of already collected data. No patient direct interaction was performed as part of this study.

In the Supplementary materials, we provide Supplementary Tables 2, 3 and 4, which include demographics details for our datasets.

### Reporting Summary

Further information on experimental design is available in the Nature Research Reporting Summary linked to this article.

## Supplementary information

Supplemental Materials

Reporting Summary

## Data Availability

The datasets analyzed during the current study are not publicly available: due to reasonable privacy and security concerns, the underlying EHR data are not easily redistributable to researchers other than those engaged in the Institutional Review Board-approved research collaborations in the FIND FH project.

## Supplementary information

**Supplementary information** accompanies the paper on the *npj Digital Medicine* website (10.1038/s41746-019-0101-5).

## Code availability

All code and configuration parameters are available in: https://github.com/som-shahlab/findFH_aphrodite. We develop and tested this code in R version 3.2 and higher and with the OMOP CDM V5.3.

## References

[1] Gidding SS (2015). The agenda for familial hypercholesterolemia: a scientific statement from the american heart association. Circulation.

[2] Abul-Husn NS (2016). Genetic identification of familial hypercholesterolemia within a single U.S. health care system. Science.

[3] Khera AV (2016). Diagnostic yield and clinical utility of sequencing familial hypercholesterolemia genes in patients with severe hypercholesterolemia. J. Am. Coll. Cardiol..

[4] Besseling J (2017). Selection of individuals for genetic testing for familial hypercholesterolaemia: development and external validation of a prediction model for the presence of a mutation causing familial hypercholesterolaemia. Eur. Heart J..

[5] Nordestgaard BG (2013). Familial hypercholesterolaemia is underdiagnosed and undertreated in the general population: guidance for clinicians to prevent coronary heart disease: consensus statement of the European Atherosclerosis Society. Eur. Heart J..

[6] National Collaborating Centre for Primary Care (UK). *Clinical guidelines and evidence review for familial hypercholesterolaemia: the identification and management of adults and children with familial hypercholesterolaemia*. (Royal College of General Practitioners (UK), 2011).

[7] Knowles JW, Rader DJ, Khoury MJ (2017). Cascade screening for familial hypercholesterolemia and the use of genetic testing. JAMA.

[8] Public Health Genomics. *Centers for Disease Control and Prevention* (2014). Available at: https://www.cdc.gov/genomics/implementation/toolkit/fh_1.htm. (Accessed: 9th December 2017).

[9] Obermeyer Z, Emanuel EJ (2016). Predicting the future - big data, machine learning, and clinical medicine. N. Engl. J. Med..

[10] Jordan MI, Mitchell TM (2015). Machine learning: Trends, perspectives, and prospects. Science.

[11] Ross EG (2016). The use of machine learning for the identification of peripheral artery disease and future mortality risk. J. Vasc. Surg..

[12] Deo RC (2015). Machine learning in medicine. Circulation.

[13] Breiman L (2001). Random Forests. Mach. Learn..

[14] Altman DG, Martin Bland J (1994). Statistics notes: diagnostic tests 2: predictive values. BMJ.

[15] Cook NR (2007). Use and misuse of the receiver operating characteristic curve in risk prediction. Circulation.

[16] Stone NJ (2014). 2013 ACC/AHA guideline on the treatment of blood cholesterol to reduce atherosclerotic cardiovascular risk in adults: a report of the American College of Cardiology/American Heart Association Task Force on Practice Guidelines. J. Am. Coll. Cardiol..

[17] Rajkomar A (2018). Scalable and accurate deep learning with electronic health records. *npj Digital*. Medicine.

[18] deGoma EM (2016). Treatment gaps in adults with heterozygous familial hypercholesterolemia in the United States: data from the CASCADE-FH registry. Circ. Cardiovasc. Genet..

[19] Kindt I, Mata P, Knowles JW (2017). The role of registries and genetic databases in familial hypercholesterolemia. Curr. Opin. Lipidol..

[20] Mata N (2011). Clinical characteristics and evaluation of LDL-cholesterol treatment of the Spanish Familial Hypercholesterolemia Longitudinal Cohort Study (SAFEHEART). Lipids Health Dis..

[21] Pérez de Isla L (2016). Coronary heart disease, peripheral arterial disease, and stroke in familial hypercholesterolaemia: insights from the SAFEHEART registry (Spanish Familial Hypercholesterolaemia Cohort Study). Arterioscler. Thromb. Vasc. Biol..

[22] Sturm AC (2018). Clinical Genetic Testing for Familial Hypercholesterolemia: JACC Scientific Expert Panel. J. Am. Coll. Cardiol..

[23] Safarova MS, Liu H, Kullo IJ (2016). Rapid identification of familial hypercholesterolemia from electronic health records: The SEARCH study. J. Clin. Lipidol..

[24] Bastarache L (2018). Phenotype risk scores identify patients with unrecognized Mendelian disease patterns. Science.

[25] Wang, F., Casalino, L. P. & Khullar, D. Deep Learning in Medicine—Promise, Progress, and Challenges. *JAMA Intern. Med*. (2018). 10.1001/jamainternmed.2018.7117. 10.1001/jamainternmed.2018.7117 30556825

[26] Hastie T, Tibshirani R, Friedman J (2009). The Elements of Statistical Learning: Data Mining, Inference, and Prediction.

[27] Wagstaff, K. Machine Learning that Matters. *arXiv* [cs.LG] (2012).

[28] Hripcsak G (2015). ObseRvational Health Data Sciences and Informatics (OHDSI): opportunities for observational researchers. Stud. Health Technol. Inform..

[29] Stang PE (2010). Advancing the science for active surveillance: rationale and design for the Observational Medical Outcomes Partnership. Ann. Intern. Med..

[30] Banda JM, Halpern Y, Sontag D, Shah NH (2017). Electronic phenotyping with APHRODITE and the Observational Health Sciences and Informatics (OHDSI) data network. AMIA Jt Summits Transl. Sci. Proc..

[31] Chapman WW, Bridewell W, Hanbury P, Cooper GF, Buchanan BG (2001). A simple algorithm for identifying negated findings and diseases in discharge summaries. J. Biomed. Inform..

[32] Jung K (2015). Functional evaluation of out-of-the-box text-mining tools for data-mining tasks. J. Am. Med. Inform. Assoc..

[33] Haase A, Goldberg AC (2012). Identification of people with heterozygous familial hypercholesterolemia. Curr. Opin. Lipidol..

[34] Civeira F, International Panel on Management of Familial Hypercholesterolemia. (2004). Guidelines for the diagnosis and management of heterozygous familial hypercholesterolemia. Atherosclerosis.

[35] Austin MA, Hutter CM, Zimmern RL, Humphries SE (2004). Genetic causes of monogenic heterozygous familial hypercholesterolemia: a HuGE prevalence review. Am. J. Epidemiol..

[36] Williams RR (1993). Diagnosing heterozygous familial hypercholesterolemia using new practical criteria validated by molecular genetics. Am. J. Cardiol..

[37] Norén GN, Caster O, Juhlin K, Lindquist M (2014). Zoo or savannah? Choice of training ground for evidence-based pharmacovigilance. Drug Saf..

[38] Harpaz R, DuMouchel W, Shah NH (2015). Comment on: ‘Zoo or savannah? Choice of training ground for evidence-based pharmacovigilance′. Drug Saf..

[39] Witten, I. H., Frank, E., Hall, M. A. & Pal, C. J. *Data Mining: Practical Machine Learning Tools and Techniques*. (Morgan Kaufmann, 2016).

[40] Niehaus, K. E., Banda, J. M., Knowles, J. W. & Shah, N. H. FIND FH—A phenotype model to identify patients with familial hypercholesterolemia. in *Proceedings of Data Mining for Medical Informatics Workshop 2015* (2015).

