# Supplementary material for: Finding missed cases of familial hypercholesterolemia in health systems using machine learning
Source: NPJ Digit Med. 2019 Apr 11;2:23. doi: 10.1038/s41746-019-0101-5 (PMC6550268; doi:10.1038/s41746-019-0101-5)
Supplement: Supplementary file 1 — Supplemental Materials [file 41746_2019_101_MOESM1_ESM.docx]

# **Supplementary Tables**

### *Demographics and patient characteristics tables*

|  | FH cases | | non-FH controls | |
| --- | --- | --- | --- | --- |
| n | 89 | 108 | 2,977 | 3,613 |
| Gender | Female | Male | Female | Male |
| Age (mean (sd)) | 47.8 (15.1) | 44.9 (13.9) | 50.4 (16.1) | 49.9 (14.2) |
| Most recent LDL-C (mg/dl) (mean (sd)) | 247 (59) | 241 (65) | 107 (8.12) | 109 (9.75) |
| PRE treatment LDL-C (mg/dl)(mean (sd)) | 259 (74)* | 257 (62)* | NA | NA |
| Race (%) |  |  |  |  |
| Asian | 10 (11.2) | 15 (13.9) | 335 (11.2) | 502 (13.9) |
| Black or African American | 2 (2.2) | 0 (0.0) | 67 (2.2) | 0 (0.0) |
| Native Hawaiian or Other Pacific Islander | 1 (1.1) | 1 (0.9) | 33 (1.1) | 33 (0.9) |
| Not specified | 27 (30.3) | 36 (33.3) | 903 (30.3) | 1,204 (33.3) |
| White | 49 (55.1) | 56 (51.9) | 1,639 (55.1) | 1,873 (51.9) |
| Ethnicity (%) |  |  |  |  |
| Hispanic or Latino | 8 (9.0) | 7 (6.5) | 268 (9.0) | 234 (6.5) |
| Not specified | 15 (16.9) | 11 (10.2) | 502 (16.9) | 368 (10.2) |
| Not Hispanic or Latino | 66 (74.2) | 90 (83.3) | 2208 (74.2) | 3,011 (83.3) |
| Had CVD Event (MI, IS, PCI/CABG) | 7(7.86) | 8(7.5) | 178 (5.98) | 241 (6.68) |
| **Supplementary Table 1**: Demographics of FH true positive cases and age and gender matched non-FH patients from Stanford dataset. * Pretreatment LDL-C was available on 59 female and 71 male cases | | | | |

| n | 13,130 | 22,432 |
| --- | --- | --- |
| Gender | Female | Male |
| Age (mean (sd)) | 69.5 (18.5) | 72.6 (12.8) |
| Race (%) |  |  |
| American Indian or Alaska Native | 50 (0.4) | 64 (0.3) |
| Asian | 1,749 (13.3) | 2,978 (13.3) |
| Black or African American | 939 (7.2) | 872 (3.9) |
| Native Hawaiian or Other Pacific Islander | 210 (1.6) | 257 (1.1) |
| Not specified | 2,926 (22.3) | 4,577 (20.4) |
| White | 7,256 (55.3) | 13,684 (61.0) |
| Ethnicity (%) |  |  |
| Hispanic or Latino | 1,720 (13,1) | 1,878 (8.4) |
| Not specified | 1,015 (7.7) | 2,009 (9.0) |
| Not Hispanic or Latino | 10,395 (79.2) | 18,545 (82.7) |

**Supplementary Table 2**: Demographics of Stanford patients that we used to find FH candidates using the Stanford model.

| n | 137 | 99 |
| --- | --- | --- |
| Gender | Female | Male |
| Age (mean (sd)) | 58.9 (17.2) | 64.6 (15.6) |
| Highest LDL-C in EHR (mean (sd)) | 168.5 (53.0) | 169.3 (48.2) |
| POST treatment LDL-C (mean (sd)) | 148.9 (60.7) | 126.5 (54.6) |
| Race (%) |  |  |
| Black or African American | 4 (2.9) | 3 (3.0) |
| White | 133 (97.1) | 96 (97.0) |
| Ethnicity (%) |  |  |
| Hispanic or Latino | 2 (1.5) | 0 (0.0) |
| Not Hispanic or Latino | 135 (98.5) | 99 (100.0) |

**Supplementary Table 3**: Demographics of FH genetically confirmed true positives from Geisinger dataset.

| n | 148 | 82 |
| --- | --- | --- |
| Gender | Female | Male |
| Age (mean (sd)) | 64.3 (15.6) | 56.9 (14.9) |
| PRE treatment LDL-C (mean (sd)) | 226.8 (94.4) | 212.0 (119.1) |
| POST treatment LDL-C (mean (sd)) | 186.8 (93.7) | 162.1 (90.6) |
| Race (%) |  |  |
| Asian | 1 (0.7) | 1 (1.2) |
| Black or African American | 1 (0.7) | 2 (2.4) |
| Other Race | 1 (0.7) | 0 (0.0) |
| White | 145 (98.0) | 79 (96.3) |
| Ethnicity (%) |  |  |
| Hispanic or Latino | 4 (2.7) | 3 (3.7) |
| Not Hispanic or Latino | 144 (97.3) | 79 (96.3) |

**Supplementary Table 4**: Demographics of FH Dutch Lipid Clinic Network (DLCN) criteria confirmed true positives from Geisinger dataset.

| n | 2484 | 2516 |
| --- | --- | --- |
| Gender | Female | Male |
| Age (mean (sd)) | 63.2 (16.3) | 67.7 (13.4) |
| Race (%) |  |  |
| American Indian or Alaska Native | 7 (0.3) | 0 (0.0) |
| Asian | 3 (0.1) | 3 (0.1) |
| Black or African American | 23 (0.9) | 24 (1.0) |
| Native Hawaiian or Other Pacific Islander | 3 (0.1) | 1 (0.0) |
| Other Race | 0 (0.0) | 1 (0.0) |
| Unknown | 3 (0.1) | 2 (0.1) |
| White | 2445 (98.4) | 2485 (98.8) |
| Ethnicity (%) |  |  |
| Hispanic or Latino | 28 (1.1) | 13 (0.5) |
| Not Hispanic or Latino | 2456 (98.9) | 2503 (99.5) |

**Supplementary Table 5**: Demographics of FH controls from Geisinger dataset.

| **Dutch Lipid Clinic** | | | ***Probability Group*** | | | |
| --- | --- | --- | --- | --- | --- | --- |
|  |  |  | **0.60 to 0.69**  **(n = 10)** | **0.70 to 0.799**  **(n=16)** | **0.80 to 0.89**  **(n=18)** | **0.90 to 0.99**  **(n=56)** |
| **Definite** | **MEDPED** | No |  |  |  | 1 |
| **Probable** | **MEDPED** | No |  | 1 | 1 | 4 |
|  |  | Yes |  | 0 | 1 | 3 |
| **Possible** | **MEDPED** | No | 1 | 8 | 7 | 34 |
|  |  | Yes | 0 | 0 | 0 | 5 |
| **Unlikely** | **MEDPED** | No | 9 | 7 | 9 | 9 |

**Supplementary Table 6**: Chart review analysis by probability group.

# **Supplementary Methods:**

### **Random Forest Classifier Error Analysis:**

|  | AUROC | PPV | Sensitivity | Specificity | F1-Score | TP | TN | FP | FN |
| --- | --- | --- | --- | --- | --- | --- | --- | --- | --- |
| Stanford Model | 0.943 | 0.882 | 0.750 | 0.998 | 0.811 | 15 | 1185 | 2 | 5 |


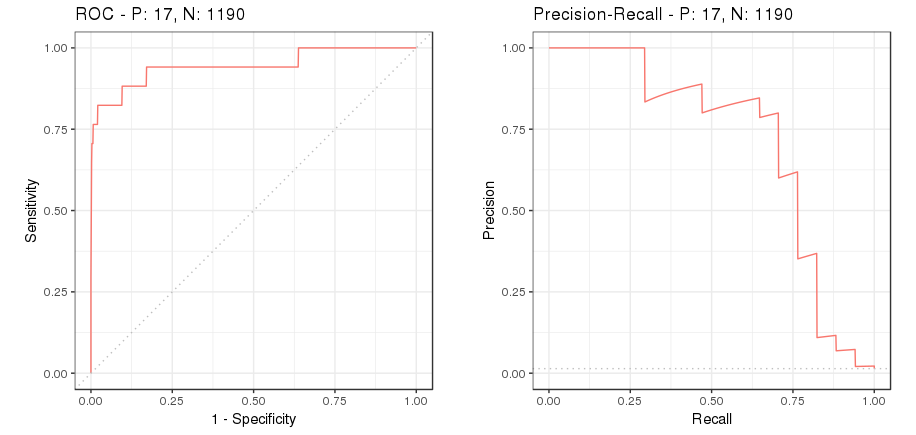


**Supplementary Table 1**. Plot for Area under ROC curve (left) and Area under PR Curve (left).
